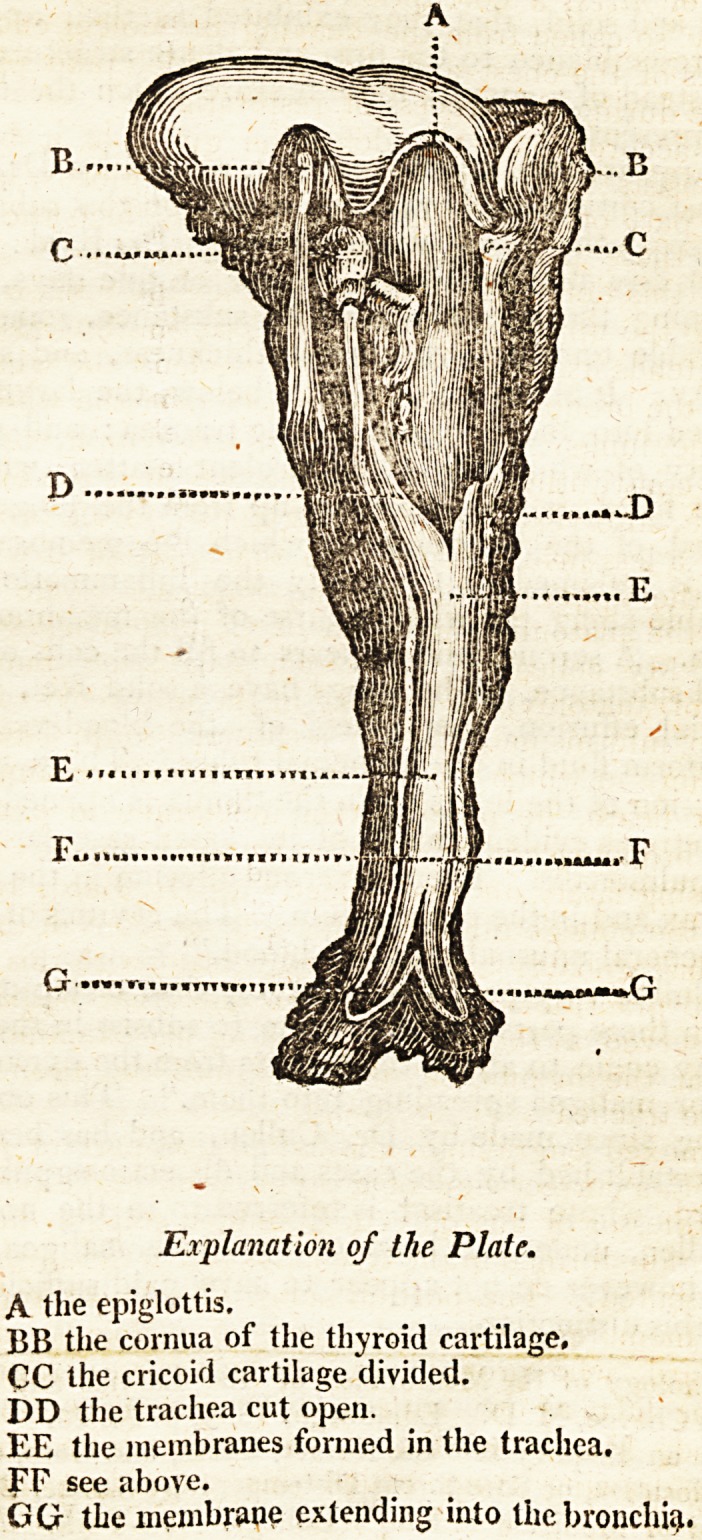# Observations on Croup or Hives, in a Letter to A. R. Delile, M.D.
*These "Observations" on a violent and fatal disease, were originally printed in the American Medical and Philosophical Register, and reprinted in a separate pamphlet at New York. Having received a copy from their ingenious author, Dr. Hosack, we present it to our readers as a valuable document on an important subject. We take this opportunity of acknowledging how much we are indebted to our brethren on the other side the Atlantic, for thus communicating their researches, which are interesting to Europeans, not only from their intrinsic value, but from the peculiar cast of their inquiries, induced by the original materials which the accident of situation constantly opens to them.—Editors.


**Published:** 1812-03

**Authors:** David Hosack


*For the Medical and Physical Journal.*

### OBSERVATIONS *on* CROUP *or* HIVES,* *in a* LETTER *to* A. R. DELILE, M.D.

*New York, June 28th,* 1811.

DEAR SIR,

YOU some time since requested me to communicate to you in writing the observations I had expressed in conversation relative to the nature of Croup, and the mode of treatment which I had found most successful in that disease. I now comply with your request, but shall confine my remarks chiefly to those points in which my views of this subject may differ from those usually met with in medical writings.

The various names under which this disease is described by authors are familiarly known to you.

---

* These "Observations" on a violent and fatal disease, were originally printed in the American Medical and Philosophical Register, and reprinted in a separate pamphlet at New York. Having received a copy from their ingenious author, Dr. Hosack, we present it to our readers as a valuable document on an important subject. We take this opportunity of acknowledging how much we are indebted to our brethren on the other side the Atlantic, for thus communicating their researches, which are interesting to Europeans, not only from their intrinsic value, but from the peculiar cast of their inquiries, induced by the original materials which the accident of situation constantly opens to them.—EDITORS.

 I look

In common language it also receives different appellations: In Ireland it is called *chock*, or stuffing; in England and Scotland, croup; but more usually in this country it receives the name of *hives*, a corruption of the term heaves, which is probably so called from the heaving or violent efforts of the muscles of the chest and abdomen, which take place in this disease during the process of respiration.

Croup, according to the definition given of it by Dr. Cullen, consists " in an inflammation of the glottis, larynx, or upper part of the trachea, whether it affects the membranes of these parts or the muscles adjoining." In one particular this definition is defective, as the disease is not confined to the upper portion of the trachea, but also most usually extends itself throughout the whole of the windpipe, even into the bronchiæ, and to a degree over the whole surface of the lungs. The effusion of the lymph, or other materials, constituting the membrane, which is the effect of this disease, also very frequently extends into the bronchiæ, though of a less firm texture than that part of it which is found in the upper portion of the trachea. Some preparations in the anatomical museum of Columbia college shew this fact. My colleague, Mr. John Augustine Smith, the professor of anatomy and surgery in the university of New-York, also informs me, that in a case of croup, lately met with by him, in which he was called upon to examine the parts after death, he observed the membrane to extend as far as the bronchiæ could be traced by the knife.

Conversing lately on this subject with Dr. Bard, the president of the college of physicians and surgeons of this city, and who has probably been more conversant with this disease than most practitioners, he informs me, that he has commonly observed, in those cases which he has examined after death, that the membrane extended into the bronchiæ as well as the trachea.

The annexed engraving exhibits, in that part of it above the line FF, a correct view of a preparation in the possession of the professor of anatomy in Columbia college, in which the membrane formed in the trachea is preserved in the manner represented. The portion below the line FF represents the same membrane extended into the bronchiæ, as observed by Dr. Bard, who pronounces it to be a correct representation of the fact, as met with by him upon dissection. To my friend, Mr. Inderwick, I am indebted for the very beautiful drawing from which this engraving has been made.

*Explanation of the Plate.*

A the epiglottis.

BB the cornua of the thyroid cartilage.

CC the cricoid cartilage divided.

DD the trachea cut open.

EE the membranes formed in the trachea.

FF see above.

GG the membrane extending into the bronchia.

Dr.

Dr. Bard also remarks, that the disease is not even limited to the trachea and bronchiæ, but that the lungs, throughout their whole substance, to a certain degree participate in the affection; insomuch that he has seen those organs rendered so dense and solid, that they exhibited in their appearance a great resemblance to the firm and dense structure of the liver, instead of a spongy loose texture which the lungs naturally present.

The appearances upon dissection, related by Dr. Cheyne in the last edition of his valuable work* on this subject, correspond with the observations made by Dr. Bard: "When the child dies after an illness of four or five days, there is found lining the windpipe a white substance, sometimes of considerable tenacity, varying in thickness, and somewhat in density. It arises at, or a little below, the larynx, and is prolonged into the divisions of the trachea: and generally a quantity of white fluid like purulent matter, with which they are filled, is seen working up from the lungs. The inner coat of the windpipe to which the membrane is attached, is inflamed. Generally the inflammation is also discernable along the whole course of the membrane of the bronchia. A serous fluid appears to fill the cells of the interstitial substance. The lungs have a solid feel, from the interstitial effusion, the fulness of the blood-vessels, and the puriform fluid in the bronchial tubes. There is little or no recession of the lungs when the thorax is opened. There are sometimes evident marks of increased vascularity in the pleura pulmonalis. There is serous effusion in the cavity of the thorax and in the pericardium. The cavities of the heart are in general unusually full of blood."

Dr. Cullen very properly observes, that croup may arise, "first in these parts, and continue to subsist in them alone, or it may come to affect these parts from the cynanche tonsillaris or maligna spreading into them." This observation was long since made by Dr. Cullen, and has been abundantly established by the cases and dissections published by Dr. Bard, whose treatise† is referred to in the nosology of Dr. Cullen, under the head of cynanche maligna. Other writers however do not appear to have paid sufficient attention to this distinction.

---

* Pathology of the Membranes of the Larynx and Bronchia. By John Cheyne, M. D. Edinburgh, 1809, p. 24, 25.

† See an Enquiry into the Nature, Cause, and Cure, of the Angina Suffocativa, or Sore-throat Distemper: by Samuel Bard, M.D. Professor of Medicine in King's College, New-York. *New-York,* 8vo. 1771.—See also American Philosophical Transactions, vol. i. p. 388.

Some

Some years since I was called in consultation to a case similar to those described in the valuable treatise of Dr. Bard. The disease began with an inflammation of the tonsils, but was soon succeeded by ulceration, attended with fœtid breath and a foul appearance of the parts affected. About the third day the inflammation extended into the trachea, producing the laborious respiration, and hoarse hollow-sounding cough, which characterise idiopathic croup; in twenty-four hours it proved fatal. The attending physician informed me, that during the first three days the child had not manifested any symptoms denoting croup; but, as in the cases recorded by Dr. Bard, they were probably induced by the inflammation and subsequent acrid secretion extending from the tonsils into the trachea. Since that time I have met with several instances of a similar nature succeeding to malignant sore-throat. Other practitioners in this city, who have had frequent opportunities of seeing croup, confirm the observation that this termination of cynanche maligna is not an unfrequent occurrence. Dr. Bard informs me, that since the publication of his Essay in 1771, he has frequently observed this disease as the sequela of cynanche maligna. Two cases of croup supervening as an accessory disease in ulcerated sore-throat are also related by Dr. Ferriar in his valuable paper on that subject. "Though there were large ulcerations in the tonsils," he observes, "there was nothing uncommon in the symptoms till the inflammation extended to the trachea, when faint, shrill coughing, hissing respiration, and restlessness, came on, which were soon followed by death."*

Croup also, in some instances, is the attendant upon scarlatina. A case of this kind occurred in a child of Mr. Peter P. Goelet, of this city: in that case ulcers of the tonsils, which were attended with considerable inflammation, and an acrid offensive discharge, preceded the symptoms of croup; but, by the use of emetic medicine, the patient was relieved of these alarming symptoms, and, by the use of bark and yeast, which were afterwards administered, both internally and as a gargle, completely restored. In Mr. Cheyne's treatise before referred to, a case of scarlet fever is recorded which proved fatal, in which the membrane was actually formed as in croup, and was removed after death by Dr. Rollo, surgeon of the Woolwich hospital.†

In some instances, especially where ulcerations take place

---

* See Med. Hist. and Reflec. vol. iii. p. 205.

† Cheyne, p. 37.

in in the larynx, croup also succeeds to *measles.** In a case related by Dr. Cheyne, it also succeeded to the secondary fever of small-pox; and by Dr. Underwood it has been known as the attendant upon the *putrid thrush.*† Croup also, says Dr. Cheyne, very often supersedes a common catarrhal affection. In a singular instance, Dr. Ferriar also observes, that he has seen pneumonic inflammation converted into a croup on the tenth day of the disease.‡ Dr. Rush remarks, "I have seen it accompany as well as succeed the small-pox, measles, scarlet fever, and apthous sore-throat. In the late Dr. Foulke it succeeded acute rheumatism. The late Dr. Sayre informed me he had seen it occur in a case of yellow fever in the year 1798."§

With these facts before us, therefore, there appears to be just ground for dividing this disease into two species: viz. *idiopathic* and *symptomatic* croup: *idiopathic* where the disease is primarily and exclusively seated in the trachea, bronchiæ, and surface, of the lungs; *symptomatic*, where it is the consequence of other previous diseases.

It is asserted by some writers, but denied by others, that cynanche trachealis is an *infectious* disease.

As the cynanche maligna and scarlatina are communicated by contagion or infection, doubtless they may also be so in their consequences; and in this way croup may be transferred by those diseases as the vehicle of communication. The cases related by Von Rosenstein,|| in evidence of the infectious nature of croup, were probably cases of cynanche maligna, similar to those described by Dr. Bard. We hence see the propriety of Dr. Cheyne's observation, that "when a physician has to visit more children than one, with a croupy affection, in a family or neighbourhood, he ought carefully to examine the state of *the fauces.*"¶ But, that idiopathic cynanche trachealis is infectious, I believe there can be no ground for supposing. I should as readily believe that an inflammation of the brain or of the pleura should be thus communicated, as an inflammation of the membrane lining the trachea; and I believe it may be safely asserted that the fact is otherwise. In the numerous families in which I have prescribed for this disease, I have never known it to be thus

---

* See Cheyne, p. 39.
† Diseases of Children, 4th edit. vol. i. p. 333.
‡ Med. Hist. and Reflec. vol. iii. p. 205.
§ Med. Inq. and Obs. vol. ii. p. 376, 3d edit.
|| Von Rosenstein on the Diseases of Children, translated by Sparmann.
¶ Ditto, p. 19.

communicated, communicated, either to the attendants upon the sick, or to other children, even though sleeping in the same room, and frequently in the same bed; but I have more than once been called in the same night to two children of the same family, both having been exposed to the same cause, and especially where there is a great predisposition to attacks of this disease, as is the case in particular families.

It also happens that, when a child has suffered one attack of croup, it becomes liable afterwards to repeated returns of the same complaint, and that too upon the application of much slighter causes than had induced the first invasion. The same observation, you know, is made of pleurisy, sore-throat, rheumatism, and most inflammatory complaints. I am credibly informed of a lady who has suffered twenty-one attacks of pleurisy. How much more susceptible of impressions is the sensible membrane lining the trachea, especially during infancy? But happily, as this sensibility diminishes by age, the returns of the disease become less frequent; and, when children arrive at the tenth year, it is comparatively of rare occurrence.

I have never visited a child upward of twelve years of age in this complaint, except where it had suffered previous attacks of it; yet, in some instances, as before remarked, adults are the subjects of this disease. In the winter of 1809, I was called to a lady who had lately removed to this city from the state of Virginia. She went to bed in perfect health; she was awakened by coughing, attended with pain, and a sense of burning in her throat. These symptoms were soon followed by difficult, hoarse, and laboured, respiration; her husband became very much alarmed, and called upon me between twelve and one o'clock: I found her in great distress, coughing almost incessantly, every inspiration being attended with the peculiar noise of croup. Her cough was dry, accompanied with the usual deep hollow sound, that characterises this disease in infancy. I immediately bled her freely from the arm, gave her an antimonial emetic, and applied a blister to the throat. I also left directions, that, if the difficulty of breathing should continue, to take a dose of calomel and James' powder, composed of five grains each, every two hours, and to dilute freely with warm toast-water, herb-tea, or barley-water, which are the drinks I usually direct in this disease. By these means she was relieved in a few hours. I was also in like manner called upon about three years ago to another lady attacked in a very similar manner, and who was relieved by the same means that have been enumerated in the former case.

During the year in which I resided in Virginia, in 1790–1,

I visited, in the neighbourhood of Alexandria, a man dying with every symptom characteristic of this disease. Dr. Mitchill, the professor of natural history in the university of this state, suffered a severe attack of croup in the spring of 1801, during his attendance at Washington as a member of congress. The interesting history of his case is subjoined. But there is an instance of this disease attacking the adult which can never be forgotten, as it deprived our country and the world of one of their most illustrious citizens, George Washington, late President of the United States.

Most writers have followed Mr. Home, in representing the disease as more particularly confined to maritime situations; but it is now well ascertained, that although croup is of most frequent occurrence on the sea-coast, where the air is loaded with moisture, and the changes of weather are most sensibly experienced, that it is still oftentimes met with in the interior of the country. The publications of Dr. Rush and Dr. Currie,* of Philadelphia, Dr. Stearns, of the county of Albany, in the state of New York, and Dr. Archer, of Maryland, afford evidence of this fact, for they have described the disease as it appears in places very distant from the sea. Dr. Cullen observes, that it is met with in inland countries as well as on the coast. Most usually it is ascribed to cold as its exciting cause. It is true it is frequently produced during the severe cold of winter; but, as far as I have noted its occurrence, it appears most frequently upon the approach of winter, and in the spring. I have also observed, that during severe blowing and stormy weather the cases of it are most numerous. During the summer season it is also produced by the same cause. I have frequently traced an attack of croup to the imprudent exposure of a child to the night air after a hot day, or to a stream of air to which it had been exposed in a hall or window.

This disease is described by Dr. Cullen, and by most practical writers, as consisting in an inflammation of the secreting membrane lining the trachea. But Dr. Millar,† Dr. Underwood, Mr. Field,‡ and Dr. Archer, of Maryland, describe two species of croup; one *inflammatory*, another which they denominate *spasmodic* croup. You well know, by your residence with me as a pupil of medicine, that it is a disease of very frequent occurrence in this city; yet, although I have

* Currie's View of the Diseases most prevalent in the United States of America, at different seasons of the year, with an account of the most approved method of treating them, &c.

† Millar on Asthma and Hooping-cough.

‡ Edinburgh Practice of Physic, vol. i. p. 355.

been been eighteen years a practitioner of medicine, and in that time have prescribed for many patients in this disease, I have never met with a single instance in which it assumed the spasmodic character, described by those gentlemen, unaccompanied by symptoms of local inflammation.

Dr. Bard, whose practice has been more extensive than that of any other physician of this city, informs me, that, from the year 1762 to the present time, he has never met with a case of croup that was not attended with symptoms of inflammation. Dr. Scott, of New-Brunswick, who has practised medicine with great reputation in the state of New-Jersey for about fifty years, makes a similar observation. It is true that this disease attacks the patient very suddenly, and that in its commencement the affection of the throat is frequently without pain, and is attended with very little fever, even during the first two or three hours after the attack; while the cough, peculiar noise, and labour of respiration which characterise croup, are very considerable; and to the friends, acquainted with the nature of the disease, and apprised of its dangerous consequences, very alarming. Most usually, however, in those cases in which the child is old enough to express its sensations, there is a sense of pricking, burning, or irritation, in the windpipe, sufficient to denote the seat of the disease; and such is the sensibility of the windpipe to the impressions made upon that delicate organ, that the local affection, as in the first attack of pleurisy, is out of all proportion to the general febrile excitement of the system; for neither the pulse or heat of skin are much affected during the first two or three hours of the disease. These facts, and the sudden relief which the patient sometimes obtains from the means prescribed during the first stage of the complaint, have, perhaps, led the authors mentioned to consider croup as, in some cases, a spasmodic disease of the windpipe: sometimes, too, especially when occurring in a delicate habit of body, the use of the common domestic remedies, viz. warm bathing and warm drinks, are sufficient, by the relaxation they induce in the system, to restore the suppressed excretions, and thereby to remove the irritation from the part affected. But, notwithstanding this happy termination, it does not follow that the disease is only spasmodic, and not inflammatory; for we frequently see catarrh, and sometimes even incipient pleurisy, by all acknowledged to be diseases exclusively of an inflammatory nature, removed without having recourse to the more active remedies usually resorted to: but unhappily this disease generally attacks children of the most robust habit of body; and, if not immediately arrested, terminates

 in in violent inflammation, accompanied with fever, which are only to be removed by the most prompt and decisive practice. In cases of this sort, to trust to the prescriptions ordinarily directed for the removal of the most violent spasmodic affections, is to do nothing; it is worse than nothing; for, while the physician temporises, the child perishes. Many lives, I believe with Dr. Ferriar, "have been sacrificed to the imaginary powers of assafœtida, or small repeated doses of antimonials, from unfounded theories of spasmadic constriction attending the disease."*

Dr. Cullen observes, that the antiphlogistic regimen is necessary in every stage of the disease, and that he has not found antispasmodic medicines of any use. It is, therefore, most safe for us to consider, with Dr. Rush, that all the varieties which this disease assumes, "are the effects of a difference only in its force or in its duration," and, to continue to use the language of that accurate clinical observer, that "they all depend upon one remote and one proximate cause." It also fortunately happens, that the practice which is found most effectual in inflammatory croup, is not opposed to that which would be indicated if the disease were exclusively spasmodic; on the contrary, the remedies found most useful in counteracting inflammation, are also among the most powerful antispasmodics. This leads me to add some further remarks on the

TREATMENT OF CROUP.

Writers upon this subject differ as widely as they do about the nature or character of the disease; but none, in my opinion, appear to have sufficiently discriminated between the different stages, in which the remedies they severally recommend ought to be employed; even Dr. Cheyne's late valuable work, and which contains the best pathology of this disease, is in some degree defective in this respect. I have been led at the bed-side to distinguish *three* distinct stages in croup: the first may be denominated the *forming stage* of the disease; in this the affection is local; the irritation has not yet extended to the whole system; the child even sits laughing and playing upon the lap of its mother, manifesting a very unusual but morbid degree of exhilaration; its skin is cool and moist, its pulse not perceptibly accelerated; but its hoarse hollow sounding, and frequently returning cough, its wheezing inspiration, its restlessness, and especially its cries after a fit of coughing, all denote, to the physician and parent acquainted with the disease, the consequences that will soon ensue, if active means be not employed

* Med. Hist. and Reflec. vol. iii. p. 210.

ployed to prevent the *second* or *febrile stage.* In this stage the whole system partakes of the irritation; the pulse is frequent, the skin hot and dry, the respiration hurried, the tongue covered with the usual white fur indicative of inflammation, the lips and cheeks remarkably florid, the cough frequent, but attended with a more acute sound than that of the first stage; every inspiration too is attended with more uniform wheezing than that which appears in the first, when occasionally an interval occurs in which the child breathes as if in health. But in this second stage no such interval is perceived; the trachea, bronchiæ, and lungs, become so surcharged by the circulating fluids, that the child has not even a momentary relief from its oppression; and in a short time, if left to itself, especially if the patient be plethoric, the countenance exhibits a purple, livid color, not unlike that of apoplexy, and is even attended with a degree of stupor, or propensity to sleep. This loaded state of the lungs and interruption to the free return of blood from the head I have frequently witnessed in this stage of croup: if the patient be now neglected, or the evacuations be sparing and insufficient, an effusion from the exhalent vessels opening into the windpipe, bronchiæ, and surface of the lungs, inevitably takes place. In the two former, the effused matter assumes a membranous appearance, probably owing to the forcible passing and repassing of the air through those preternaturally constricted tubes; but in the lungs themselves it appears in the form of a viscid fluid, partly resembling both phlegm and pus. When this effusion has actually taken place, the febrile symptoms sensibly abate, and sometimes disappear altogether; the child is also apparently free from pain, but it suffers violent paroxysms of cough and difficult breathing, attended with an irregular and spasmodic respiration, as in asthma, or dropsy of the chest, and with similar intervals of ease. These paroxysms, in young children, continue but a few hours before dissolution. But, in children arrived at eight or ten years of age, they frequently continue several days. A daughter of Gen. Morton, whom I saw in consultation, continued to struggle with those painful paroxysms at least four or five days after the febrile stage had terminated, and the effusion of matter, constituting the membrane, was supposed to have taken place. In some cases the impediment to inspiration, and the distress attending the paroxysms are so great, that the only position in which the patient can respire, is with the head thrown back. In this situation the trachea is extended, and thereby its capacity increased, and adapted to the membrane which it encloses. In some instances before death, general convul-

 sions sions ensue, which speedily terminate the sufferings of the patient. This stage, in which the membranous effusion takes place, I denominate the *membranous* or *purulent stage:* from this advanced state of the disease recovery is so rare, that it is not to be expected; it might almost be denominated the *fatal* stage of croup. These distinctions it is, in my opinion, important for the practitioner to keep in view, as they lead to important conclusions in practice.

They teach us, during the first or forming stage of this disease, to adopt the most active means of restoring the suppressed secretions of the trachea and surface of the lungs, and by open bowels and perspiration to guard against the general excitement of the system. For this purpose, when called to a patient labouring under the first symptoms, in which the disease appears to be confined to the parts primarily affected, it is my practice to administer an emetic composed of tartarised antimony and ipecacuanha: to a child under two years of age I direct from one to two grains of the emetic tartar, with from five to ten grains of ipecacuanha every fifteen minutes, until it operates to such a degree as to induce a plentiful secretion from the trachea and lungs. It is surprising, in some instances in this disease, to see the immense quantity of viscid ropy phlegm discharged by the operation of an active emetic at this period of the complaint; but when this discharge has been accomplished, and the cough has become loose, which is an evidence of the natural secretion being restored upon the surface of the parts affected, we may, in most cases, consider the patient secure from danger.

It is usual with many physicians, upon these occasions, to administer large quantities of warm water to the patient, under the operation of an emetic: this practice, in my opinion, by washing the medicine out of the stomach, and diluting it, diminishes the nausea and general relaxation which it otherwise produces, and upon which its beneficial effects in a great degree depend. When the emetic has no other effect than to produce vomiting, I immediately direct the bowels to be emptied by the common domestic injection, and a dose of calomel from five to ten grains to be given, unless the child may be completely relieved; for it frequently happens that an emetic alone, by restoring the excretions from the windpipe and lungs, and the other evacuations by perspiration and stool which it creates, affords immediate relief, especially if the physician be called early in the disease.

The same result is thus noticed by Dr. Rush, in his excellent practical remarks on cynanche trachealis:

"In

"In the forming state of this disease, which may be easily known by a hoarseness, and a slight degree of stertorous cough, a puke of antimonial wine, tartar emetic, ipecacuanha, or oxymel of squills,* is for the most part an immediate cure. To be effectual it should operate four or five times. Happily children are seldom injured by a little excess in the operation of this class of medicines. I have prevented the formation of this disease many hundred times, and frequently in my own family, by means of this remedy." †

But it too frequently happens, that many of the common family prescriptions are in the first instance employed, and much valuable time lost before the physician is called upon; in that case, if the febrile symptoms have already manifested themselves, other remedies are indicated. In this second stage of croup, such is the determination of the circulating fluids to the part affected, and such the general febrile excitement of the system, that the most efficient means of diminishing the plethora of the blood-vessels, and of diverting the irritation from the part affected, become necessary. With this view, the patient should be bled freely, in proportion to its age and powers of constitution; say for a child under two years, from two to four ounces; from two to six years, from four to six or eight ounces; and to be repeated as the urgency of the symptoms may require. Most writers recommend the blood to be taken from the jugular veins: as I have never, even in the youngest children, experienced any difficulty in opening a vein upon the back of the hand, and of drawing a sufficient quantity of blood from that part, especially after immersing the hand a short time in warm water, I have never had occasion to open a vein in the neck; and as the child is generally very restless in this disease, and there is on this account more hazard in opening one of the jugular veins than those on the back of the hand, I have uniformly preferred the latter. It is also preferable on other accounts: it is difficult to ascertain the quantity of blood drawn from the jugular; the vein cannot be so readily closed, and the orifice is apt to open afresh by a violent fit of coughing. I confess I read with surprise the observation of Dr. Cheyne, that it is difficult to procure a sufficient

* As the operation of the squills is very much limited to the stomach, and does not produce the same general relaxing effects upon the whole system that are produced by antimony and ipecacuanha, and having frequently been altogether disappointed in the emetic effects of it, I have totally abandoned the use of this medicine in the first stages of this disease.

† See Med. Inq. and Obs. vol. ii. p. 377. 3d edit. 1809. Philad.

quantity quantity of blood from any other than the jugular vein. Dr. Ferriar makes a similar remark, "that in the case of very young children, we must almost despair, for it is extremely difficult to procure any blood from them by the lancet." These difficulties I have never experienced; the vein on the back of the hand, even in children six weeks old, being always perceptible to the finger, if not to the eye.

Although I am not an advocate for small bleedings in croup, let me here take occasion to express my disapprobation of the practice of some physicians, especially that recommended by the late Dr. Bayley, of this city, Dr. Ferriar, of Manchester, and Dr. Dick, of Alexandria;* I mean that of bleeding the patient until fainting be induced. The relaxing effects of blood-letting upon the system are no doubt desirable in this complaint, and were probably the objects which the advocates of this mode of treatment had in view; but having observed, in some instances, very serious and permanent evils to the constitution, occasioned by the debility which this profuse evacuation had produced, and knowing that even the most violent attacks of croup will yield to a less excessive evacuation by the lancet, when conjoined with other remedies, I have hitherto objected to this practice in the extent it has been recommended. After blood-letting generally some partial relief is immediately obtained; respiration is less frequent; the peculiar noise of inspiration is also diminished; the cough becomes more loose and yielding; the skin is rendered moist; and the pulse less tense and frequent. But these favorable symptoms are oftentimes deceptive, and of short duration: the cough, laboured respiration, and heat of skin, are perhaps all renewed in the course of an hour. In that case the antimonial emetic must be immediately employed. Although the force of the disease may have been greatly subdued by blood-letting, the alarming symptoms so frequently return, that I am now in the constant practice of prescribing the emetic immediately after blood-letting has been performed, without waiting to ascertain the effects which the bleeding alone might produce; if, however, after the operation of the emetic, the symptoms still continue violent, I usually repeat the bleeding, immerse the patient in a warm bath, apply a large blister to the throat covering the larynx and trachea, and administer a cathartic of calomel, from five to

---

* See 3d Supplement to Dr. Barton's Med. and Physical Journal, for May, 1809. p. 242.

ten ten grains,* repeating this medicine every two hours, until it produces some sensible effect in this respect, at the same time soliciting its operation upon the bowels by injections occasionally administered.

These several remedies having been employed, and having failed completely to subdue the febrile symptoms, and to divert the irritation from the trachea and lungs, I next direct small doses of calomel and James' powder, from two to five grains of each, to be given every two hours to a child under four years of age; but when sufficient evacuation from the bowels may have been procured, I frequently prescribe the antimonial wine, or a solution of tartar emetic, in such doses as to excite a considerable degree of nausea and relaxation; with these I occasionally blend a small portion of laudanum, where it may be indicated either in consequence of the profuse evacuation by the bowels, or when the cough may be very harassing to the patient, which is sometimes the case when the febrile symptoms are greatly moderated; in other respects laudanum should be administered with great caution in this disease.

The physician is sometimes called upon at a late period of the disease, where the means which have been described have not been employed; or, if they have been, may not have succeeded; and in which the third stage of the disease

* Such is the efficacy of calomel in the treatment of croup, that some practitioners place their chief dependence upon it in every stage of this disease, even in its most violent forms. Dr. Stearns, of Albany, a physician of great reputation, and who is said to have been singularly successful in the cure of croup, prescribes it in connection with the cerated glass of antimony, at the same time administering a decoction of the seneka snake-root, (*polygala senega*): for a child of a year old, when the disease has assumed its most alarming symptoms, he directs 20 grs. of calomel, with 8 grs. of the cerated glass of antimony; for a child of two years of age, the dose is increased to 25 or 30 grs. of calomel, with a proportionate increase of antimony. This combination, Dr. Stearns observes, generally operates two or three times as an emetic, and as often by stool; but if the disorder continues after the operation of this dose, he gives the decoction of seneka, and at the expiration of every eight hours repeats the dose of calomel and antimony, until the cure is complete. In common cases he remarks, that one dose is sufficient, and that he has never found it necessary to give more than four. Dr. Stearns, considering croup to arise from a torpor in the absorbents of the trachea, and not primarily an inflammatory affection, disapproves of blood-letting, "as a very hazardous remedy, and which ought never to be prescribed in simple cases of croup."—*See Coxe's Med. Museum*, vol. v, p. 195.

has become apparent. Respiration, as in the two preceding stages, is still laborious, accompanied with the same wheezing noise upon every inspiration ; the cough also continues violent, without the least expectoration, and returns in paroxysms, in which the patient is threatened with immediate suffocation ; the countenance exhibits a bluish livid appearance, at the same time that the patient manifests the greatest anxiety and distress ; occasionally, however, it has intervals of ease ; in which its sufferings are apparently inconsiderable ; but these intervals are of short duration, and afford no prospect of relief, for the effusion before mentioned, and the consequent formation of a membranous matter lining the trachea and bronchiæ has already taken place. In this stage of the disease, it has occasionally happened that portions of the membrane have been thrown off by coughing, by which the patient has happily been preserved. Two cases of this kind are related by Dr. Home, (p. 53, 54,) which have induced him to hope that " art, though not in the way of internal medicine, may attempt effectuating the same end."

But, although nearly fifty years have elapsed since the publication of Dr. Home's treatise, in which this suggestion is contained, we do not learn that in a single well authenticated case the operation of opening the trachea has been successfully performed; and when we recollect what has already been stated, that the disease is not limited to the trachea, that the inflammation and effusion of matter are spread over the greater part of the surface of the lungs, that the membrane itself frequently extends below the division of the trachea, the inference is plain that even, if the membrane alone could be detached, it would still be doubtful how far the disease would be removed by the operation. In one case related by Dr. Home, " part of the membrane was thrown up, yet the patient died," (p. 53.) But, although it were certain that the membrane was confined to the trachea alone, such must be the difficulty of detaching it from its connection, and such the embarrassments, from the restlessness of the child, the constant movement of the larynx in respiration, the discharge of blood, &c. that must necessarily attend an operation of this sort, that I should be inclined to rest the whole hopes of relief, even in this advanced stage of the disease, upon the use of internal medicines.

Calomel, in small but repeated doses, squills, the syrup of onions, the seneka snake-root, ammoniac, and assafœtida, and the vapour of vinegar and water, are the medicines upon which I am inclined to place most reliance at this advanced period of croup ; as they are a class of remedies calculated to excite the secretion from the lungs, without impairing the general powers of the system, they afford, if steadily persisted in, the best means of loosening and of ejecting the membranous matter, as well as the fluid materials effused over the surface of the lungs.

The following case, related by Dr. Rush, of the good effects of calomel in the advanced stage of croup, should incite us to the diligent use of this remedy, even after the effusion of the matter constituting the membrane has been ascertained to have taken place. The doctor observes, "I once attended a man from Virginia of the name of Bampfield, who, after an attack of this disease, was much distressed with the stertorous breathing and cough, which belong to it: I suspected both to arise from a membrane formed by inflammation in his trachea. This membrane I supposed to be in part detached from the trachea, from the rattling noise which attended his breathing. He had used many remedies for it to no purpose. I advised a salivation, which in less than three weeks perfectly cured him."*

But these stimulant remedies, excepting calomel, the use of which, in the first stages of croup, has already been noticed, should, in my opinion, be confined to the third stage of this disease. Many families in this city, and some physicians too, are in habits of prescribing the syrup of onions in all stages of croup, without discrimination. So powerful a stimulant cannot certainly be administered with safety where blood-letting and other means of reducing the increased excitement of the system are indicated. Dr. Archer, of Maryland, has rendered an important service to medicine by introducing into general use the polygala senega, as a remedy in croup. Hitherto, however, he has certainly disappointed the expectations of most practioners, because it has been prescribed indiscriminately in every stage of the disease; whereas, for the very reason that it is so useful in exciting the vessels of the trachea and lungs to a powerful excretion of the materials oppressing them in the last stage of croup, it is certainly a hazardous prescription when those organs are preternaturally excited, as they are both in the forming and febrile stages of this disease. I subjoin Dr. Archer's formula for preparing and administering this medicine: he observes,

"The decoction of the root is the manner in which I have generally seen it used; the strength must be determined by the physician: it must be so strong, as to act sensibly on his own fauces, in exciting coughing, &c. Half an ounce of the

* Med. Inq. and Obs. vol. ii. p. 380.

root of seneka, bruised and simmered in a close vessel in half a pint of water, until reduced to four ounces, will probably in most cases be sufficiently strong. A tea-spoonful of this to be given every half hour or hour, as the urgency of the symptoms may demand; and during these intervals a few drops occasionally, to keep up a sensible action of the medicine in the fauces, until it acts as an emetic or cathartic; then repeated in small quantities, and so frequently, as to keep up a constant stimulus in the mouth and throat." (p. 33, 34.) "The powder," he adds, "has lately been used in doses of four or five grains, mixed in a little water, with effects equally pleasing as the decoction."

For the same reason that stimulant remedies are thus indicated, blood-letting, emetics, the warm bath, and such other medicines as relax and debilitate the system, and which were indicated during the two first stages, ought in this to be prohibited; for in this debilitated state of the system they not only diminish the power of secretion, but of ejecting the matter secreted. If circumstances, however, should indicate an emetic in this stage of the disease, and the decoction of seneka should prove insufficient, the sulphate of zinc or copper is certainly preferable to that of antimony or ipecacuanha, the former being less debilitating, while they afford all the advantage which can be obtained from the mechanical operation of vomiting, and which is all that can be desired at this advanced period of the disease; at this time it is also necessary to sustain the strength of the patient by more nutritious food than is proper in the first stages: a cup of sago, arrow-root, chicken soup, or weak wine whey, are now indicated; but the latter should be carefully abstained from during the inflammatory stages of this disease, when the patient should be confined to such drinks and nourishment as are least calculated to excite the system. Seeing then, that so little remains to be done in this third stage of croup, we are taught the importance of very active treatment during the first and second stages of this disease.

As you have had an opportunity, during the prosecution of your medical studies in this city, of witnessing the practice I have recommended, you can bear testimony to its success in those cases in which advice is called for in the commencement of the disease. Candour, however, obliges me to acknowledge, that in the course of my practice I have lost two patients in this complaint; the one in the month of September, 1797, a child of Mr. Nexsen; the other in April, 1808, a child of Mr. Herman Hendricks, of this city. Generally speaking, I consider croup in its early stage as much under the control of the remedies which have been enumerated, rated, as a pleurisy or any other inflammatory disease. But, as Dr. Ferriar justly remarks, "if the alarming symptoms are not mitigated during the first six hours, the disease will generally prove fatal."*

If the view I have taken of this interesting subject may have any claims to your attention, or be found of importance in the treatment of croup, it will afford me pleasure that I have endeavoured to comply with your request.

I am, dear Sir, with great regard,
Your's, &c.

## Figures and Tables

**Figure f1:**